# Discovery of a Carbazole-Derived Lead Drug for Human African Trypanosomiasis

**DOI:** 10.1038/srep32083

**Published:** 2016-08-26

**Authors:** Sarah M. Thomas, Andrei Purmal, Michael Pollastri, Kojo Mensa-Wilmot

**Affiliations:** 1 Department of Cellular Biology, Center for Tropical and Emerging Global Diseases, University of Georgia, Athens, Georgia 30602, USA; 2 Cleveland BioLabs, Inc., Buffalo, New York 14203, USA; 3 Department of Chemistry and Chemical Biology, Northeastern University, Boston, Massachusetts 02115, USA.

## Abstract

The protozoan parasite *Trypanosoma brucei* causes the fatal illness human African trypanosomiasis (HAT). Standard of care medications currently used to treat HAT have severe limitations, and there is a need to find new chemical entities that are active against infections of *T*. *brucei*. Following a “drug repurposing” approach, we tested anti-trypanosomal effects of carbazole-derived compounds called “Curaxins”. *In vitro* screening of 26 compounds revealed 22 with nanomolar potency against axenically cultured bloodstream trypanosomes. In a murine model of HAT, oral administration of compound **1** cured the disease. These studies established **1** as a lead for development of drugs against HAT. Pharmacological time-course studies revealed the primary effect of **1** to be concurrent inhibition of mitosis coupled with aberrant licensing of S-phase entry. Consequently, polyploid trypanosomes containing 8C equivalent of DNA per nucleus and three or four kinetoplasts were produced. These effects of **1** on the trypanosome are reminiscent of “mitotic slippage” or endoreplication observed in some other eukaryotes.

Human African trypanosomiasis (HAT) is a disease endemic to regions of sub-Saharan Africa, and is caused by the protozoan parasite *Trypanosoma brucei*. Nearly 70 million people are at risk of contracting HAT^1^. Drug therapy is necessary to cure this otherwise fatal infectious disease^2^.

The five drugs currently registered to treat HAT are suramin, pentamidine, melarsoprol, eflornithine and nifurtimox. Except for nifurtimox (administered orally, but only in combination with the injectable drug eflornithine), none are orally bioavailable. Thus, an entirely orally bioavailable treatment regimen does not exist for treatment of HAT. Other problems relating to safety have led to renewed calls for safe and orally bioavailable anti-HAT drugs, even as clinical trials for two leads (Fexinidazole^3^ and SCYX-7158^4^) are ongoing (reviewed in refs 1, 5 and 6).

There are several strategies for discovering drugs against neglected human diseases such as HAT^7^,^8^,^9^,^10^. In this study we utilize a “drug repurposing” approach^7^ in which drugs developed for one indication are tested for efficacy against a different disease. Chemical scaffolds of drugs that are active against parasites *in vitro* and in a mouse model of HAT could be subsequently optimized through medicinal chemistry efforts to create novel anti-trypanosome compounds^10^,^11^.

Carbazole scaffolds are found in some marketed drugs or are “hits” in development for treatment of chronic disease. For example, carprofen, a non-steroidal anti-inflammatory analgesic is a carbazole derivative^12^. *N*-alkyl carbazoles as well as aminopropyl-carbazoles are under investigation as lead drugs to treat Alzheimer’s and Parkinson’s diseases^13^,^14^,^15^,^16^. As part of a drug discovery initiative, Cleveland BioLabs, Inc. synthesized a class of carbazole derivatives termed “Curaxins”. Some Curaxins can intercalate into DNA, though they are non-genotoxic in that they do not induce DNA damage^17^ and influence activity of the “facilitates chromatin transcription” (FACT) complex in some human cancer cells^17^,^18^.

We tested this class of compounds against *T*. *brucei* for several reasons. First, several of them were orally bioavailable and had excellent *in vivo* toxicology properties^17^. Moreover, CBL0137 (**1**, Fig. 1a) has completed phase I clinical trials for treatment of advanced solid tumors and lymphomas^19^. Finally, methods for synthesis of this family of compounds were available, enabling production of new analogs based on evolving phenotypic structure-activity relationship data^20^.

We report here that many representatives of this class of compounds inhibited proliferation of bloodstream *T*. *brucei in vitro* at nanomolar concentrations. CBL0137 (**1**), CBL0159 (**2**) and CBL0176 (**3**) were tested in a mouse model of HAT. Administered orally, they increased survival of infected mice compared to control untreated animals. Compound **1** cured 100% of infected mice, qualifying it as a lead drug worthy of pre-clinical evaluation studies according to guidelines on tropical diseases set forth by the World Health Organization^21^. In “mode of action” studies, we found that **1** inhibits mitosis and re-licenses entry into S-phase of the cell division cycle, leading to emergence of polyploid *T*. *brucei*.

## Materials and Methods

### Drugs

Samples of compounds (>95% pure) including CBL0137 (lot #10-106-88-30) were provided by Cleveland BioLabs, Inc. (Buffalo, NY). For *in vitro* studies, 10 mM stock solutions of the compounds were prepared in dimethylsulfoxide (DMSO). For oral gavage of mice, the compounds were formulated in 0.2% hydroxypropyl methylcellulose (HPMC).

### Cell culture

Bloodstream form (BSF) *Trypanosoma brucei* was maintained at densities below 10^6 ^cells/mL at 37 °C, 5% CO_2_ in HMI-9 medium supplemented with 10% fetal bovine serum (Atlanta Biologicals; Atlanta, GA), 10% SERUM PLUS^TM^ (Sigma; St. Louis, MO), and 1% antibiotic-antimycotic Solution (Corning cellgro^®^; Corning NY)^22^. *T*. *brucei brucei* RUMP528^23^, and *T*. *brucei rhodesiense* KETRI 2482^24^ (a gift from Stephen Hajduk, University of Georgia), were used for these studies. All trypanosome experiments were performed using *T*. *b*. *brucei* unless otherwise specified.

Human HeLa cells were grown in 75 cm^2^ vented cap culture flasks (Corning) at 37 °C, 5% CO_2_ in Dulbecco’s modified Eagle’s medium (DMEM) (Corning cellgro^®^) containing 10% FBS, and 1% antibiotic-antimycotic Solution^25^. Cultures were maintained at up to 80% confluency.

### Trypanosome and HeLa cell-based proliferation inhibition assays

*T*. *brucei* and HeLa cells were cultured as described^9^ with modifications.

#### T. brucei

*T*. *brucei* (*brucei* or *rhodesiense*) were seeded at 4 × 10^3 ^cells/mL in 24-well plates (0.5 or 1 mL of culture per well). Cells were incubated for 48 h with 1 or 2 μL of DMSO (vehicle control) or compound in DMSO. Compounds were initially tested at 10 nM, 100 nM or 1 μM, to define a range where cell density responded to drug concentrations. Then, at least five drug concentrations^26^ covering this range was used for subsequent assays. Each concentration was tested in duplicate. Cell density was determined with a Neubauer Bright-Line hemocytometer (Sigma) after 48 h. Cells were counted only if they were motile. Mean cell counts were plotted against compound concentration and the GI_50_ (compound concentration that inhibits *T*. *brucei* proliferation by 50%) was determined by linear interpolation using Excel for Mac 2011 (Microsoft)^27^. Final mean GI_50_ values were calculated based on two independent experiments, each with at least five data points performed in duplicate.

#### HeLa cells

HeLa cells were seeded to 1 × 10^5 ^cells/mL in 24-well plates (1 mL of culture per well) and incubated drug-free for 24 h at 37 °C, 5% CO_2_. DMSO or stock inhibitor concentration (5 μL) was added to obtain the specified final concentrations. Cells were incubated for 24 h^28^,^29^ at 37 °C, 5% CO_2_. Next, cells were trypsinized and cell density was determined with a Neubauer Bright-line hemocytometer as described^9^. GI_50_ for each compound was calculated by plotting data for the cell counts in Excel as described for *T*. *brucei* above. Data were obtained from two independent experiments, four separate biological replicates.

### High throughput trypanosome proliferation inhibition assay

*T*. *brucei* were seeded at 4 × 10^3 ^cells/mL and dispensed into wells of a 384-well black plate (Greiner; Frickenhausen, Germany). Wells in two columns per plate were filled with 50 μL of HMI-9 medium using a Multidrop Combi (Thermo Scientific; Waltham, MA) to serve as a background control. Fifty microliters of trypanosome suspension was added to separate columns using a Multidrop Combi. Lastly, an additional 50 μL of cell resuspension was added to only the top row of wells that contained cells (row A, labeled by Greiner), using an *Xplorer* multichannel pipette (Eppendorf; Hamburg-Eppendorf, Germany) bringing final volumes of cell-containing wells in the top row to 100 μL.

Prior to their addition to assay plates, DMSO or compounds in DMSO were diluted 1:4 (v/v) with HMI-9 medium in 0.2 mL tubes (MidSci; St. Louis, MO). A microliter of DMSO (25%) or diluted drug was added to wells containing cells of the top row using an *Xplorer* multichannel pipette. Each concentration was tested in duplicate. Cells and DMSO/drug were mixed and serially diluted 1:2 down the rows of the plate.

Plates were incubated for 48 h at 37 °C and 5% CO_2_ and loaded into a Fluoroskan Ascent Microplate Fluorometer (Thermo Scientific). Cells were lysed by addition of 15 μL of SYBR Green I (Invitrogen; Carlsbad, CA) in lysis solution^30^ (5X SYBR Green I, 30 mM Tris pH 7.5, 7.5 mM EDTA, 0.012% saponin and 0.12% Triton X-100) to each well. Plates were agitated at 1200 rpm for 45 s, incubated in the dark for 1 h at room temperature, and fluorescence was detected (ex: 485 nm, em: 538 nm) using 100 ms integration time. Data collected was exported to Excel from Ascent Software (Thermo Scientific). Base-line corrected non-linear regression graphs were generated and GI_50_ values determined using GraphPad Prism (GraphPad Software; La Jolla, CA). Final mean GI_50_ values were calculated based on four separate biological replicates.

### Infection of mice with *T*. *brucei brucei*

Cultured *T*. *brucei* were pelleted and resuspended in cold phosphate buffered saline (PBS) containing 1% glucose at 1 × 10^5 ^cells/mL. Female Swiss-Webster mice (8–10 weeks old, 20–25 g, n = 4 per group) (Harlan; Indianapolis, IN) were infected intraperitoneally (i.p.) with 10^4^ bloodstream trypanosomes in 100 μL of PBS using 26G needles. To avoid damage to *T*. *brucei* cells, mechanical stress during administration was minimized by avoiding repeated pulling and pushing movements of cell resuspension through the syringe needle. Starting 48 h post-infection, and every 1–8 days thereafter, parasitemia was monitored by collecting 3 μL of blood from the tail vein of mice. Blood samples were supplemented with 21 μL of RBC Lysis Solution (Qiagen; Valencia, CA) and incubated at room temperature for 15–45 min. prior to observing for parasites using a Neubauer Bright-line hemocytometer. Mice with parasitemia greater than 2 × 10^8 ^trypanosomes/mL were euthanized. Mice were considered cured if they lived more than 30 days after experimental treatment ended and had no detectable parasites in the blood at that time^31^,^32^. To compare animal survival in treatment and vehicle control groups, Student’s *t*-test was performed in Excel to generate *p*-values. All animal experiments were conducted with the approval of the Institutional Animal Care and Use Committee (IACUC) at the University of Georgia. All experimental protocols involving animals were carried out in accordance with the approved guidelines of the IACUC at the University of Georgia.

### Drug administration and determination of parasitemia in mice

Formulated compounds or vehicle control (0.2% HPMC) were administered by oral gavage starting 24 h after mouse infection with *T*. *brucei*^33^,^34^. Animals were weighed before each compound administration, and dose volume was adjusted based on body weight (10 mL/kg) to deliver accurate doses of test compounds. The treatment was ceased for animals when normalized body weight (NBW) loss exceeded 20%, and resumed after NBW recovery above 90%. Compounds **1**–**3** were administered orally once per day of treatment 24 h post-infection as follows. **1**: 30 mg/kg or 40 mg/kg, treatment regimen of “4 days on/2 days off” for a total of 14 doses; **2**: 20 mg/kg (days 2–5), 10 mg/kg (day 7), 5 mg/kg (days 8–9), no drug (day 6, days 10–13); **3:** 25 mg/kg (days 2–5), 12.5 mg/kg (day 7), 6.25 mg/kg (days 8–10), no drug (day 6, days 11–13). Scheduled euthanasia by CO_2_ overdose followed by incision to form a bilateral pneumothorax was conducted on mice considered cured 30 days after the end of experimental treatment (see previous section). Mice with parasitemia greater than 2 × 10^8 ^trypanosomes/mL or NBW losses ≥30% were subjected to unscheduled euthanasia.

### Effects of ethidium bromide and compound 1 on kinetoplast duplication and nucleus mitosis

#### Thirty-hour compound 1 treatment of trypanosomes

*T*. *brucei* were seeded at 1 × 10^5 ^cells/mL in 175 cm^2^ vented cap culture flasks containing 100 mL of HMI-9 medium and incubated at 37 °C, 5% CO_2_ for 30 h with **1** (200 nM), ethidium bromide (EtBr, 200 nM), sterile deionized H_2_O or DMSO (0.1%). At stated times trypanosome density was determined using a Z2 Coulter Counter (Beckman). A 10 mL aliquot of cell culture per sample (1 × 10^6^–2 × 10^7 ^cells total) was collected every 6 h and analyzed using fluorescence microscopy as described below. At the same time-points a 5 mL aliquot (5 × 10^5^–1 × 10^7^ cells total) was collected and analyzed by flow cytometry as described below. All data reported were obtained from three independent experiments.

### Fluorescence microscopy

HMI-9 medium (5–10 mL) containing 1 × 10^5^–2 × 10^6 ^cells/mL of *T*. *brucei* was centrifuged (5 min, 3000 × g at room temperature) to pellet the cells. After supernatant removal cells were resuspended in 1 mL of PBS containing 4% paraformaldehyde (Affymetrix; Santa Clara, CA) and incubated for either 1 min at room temperature or up to two weeks at 4 °C. Fixed cells were adhered to poly-L-lysine coated coverslips for 15 min at room temperature. Coverslips were washed with approximately 0.1–1 mL of PBS, air dried, then mounted on 2 μL of DAPI (1.5 μM) in Vectashield (Vector Labs; Burlingame, CA). For quantitation, 150 cells per sample were counted for each independent experiment using an EVOS^®^ FL inverted fluorescence microscope (Life Technologies; Grand Island, NY). Images for figures were captured on an Applied Precision DeltaVision microscope system (GE Healthcare; Issaquah, WA) using an Olympus IX-71 inverted microscope (Olympus; Center Valley, PA) at 60X, ex: 435 nm, em: 448 nm. To compare effects on organelle DNA duplication by drug treatment and vehicle control groups, Student’s *t*-test was performed in Excel to generate *p*-values.

### Flow cytometry

*T*. *brucei* (between 5 × 10^5^ cells to 1 × 10^7^ cells) in 5 mL of culture medium were centrifuged (3 min, 3000 × g at room temperature) to pellet cells and washed once with 1 mL of PBS containing 10 mM glucose. Cells were fixed in 1 mL of 70% methanol supplemented by PBS at 4 °C for up to two weeks, centrifuged (3 min, 2000 × g at room temperature), and resuspended at 5 × 10^5 ^cells/mL in 1X PBS. RNase A and propidium iodide (PI) were added to each sample to final concentrations of 500 μg/mL and 7.5 μM, respectively, and the suspension was protected from light during incubation for 1 h at 37 °C^35^. Samples were placed on ice for 15 min and data was immediately collected on a CyAn ADP Analyzer (Beckman Coulter; Hialeah, FL). During analysis with FlowJo software (FlowJo, LLC; Ashland, OR), trypanosomes were gated from background debris by plotting “events” as forward scatter vs. side scatter (both on the logarithmic scale). Single trypanosomes were gated for cell cycle analysis from cells stuck together (“doublets”) by plotting trypanosome events as PI fluorescence vs. pulse width. Distribution of trypanosomes into groups containing different amounts of chromosomal DNA was performed using Watson-Pragmatic algorithm in FlowJo. DNA distribution values from independent experiments were averaged with error bars generated and graphed in Excel. To compare effects of drug treatment on DNA content of experimental and vehicle control groups, Student’s *t*-test was performed in Excel to generate *p*-values.

### “Delayed killing” effects after 6-h exposure of cells to compound 1

#### T. brucei cells

*T*. *brucei* were seeded at 5 × 10^5 ^cells/mL in 25 cm^2^ vented cap culture flasks containing 5 mL of HMI-9 medium and incubated 6 h with **1** (1 μM) or DMSO (0.1%). Cells were centrifuged (3000 × g, 5 min, room temperature), washed twice with HMI-9 medium and resuspended at 1 × 10^5 ^cells/mL in 5 mL of HMI-9 medium. Trypanosomes were incubated at 37 °C, 5% CO_2_ for 48 h. Cell density was determined with a Neubauer Bright-line hemocytometer as described for cell proliferation assays. Data were obtained from two independent experiments, four separate biological replicates.

#### HeLa cells

HeLa cells were seeded to 1 × 10^5 ^cells/mL in 24-well plates (1 mL of culture per well) and incubated drug-free for 24 h at 37 °C, 5% CO_2_. Next, **1** (1 μM) or DMSO (0.1%) was added and cells were treated for 6 h. Cells were washed, trypsinized, and resuspended in equivalent volumes of HMI-9 medium^9^. Cell density was determined using a Neubauer Bright-line hemocytometer as described in the cell-based proliferation assays. Samples were resuspended in HMI-9 medium to 5 × 10^4 ^cells/mL and incubated for 48 h. Cells were counted by hemocytometer, and resuspended to 5 × 10^4 ^cells/mL every 48 h for 8 days (192 h) post-treatment. Data were obtained from two independent experiments, four separate biological replicates.

## Results

### Effect of compounds on trypanosome proliferation: Preliminary structure-activity relationship (SAR)

**Figure 1:**
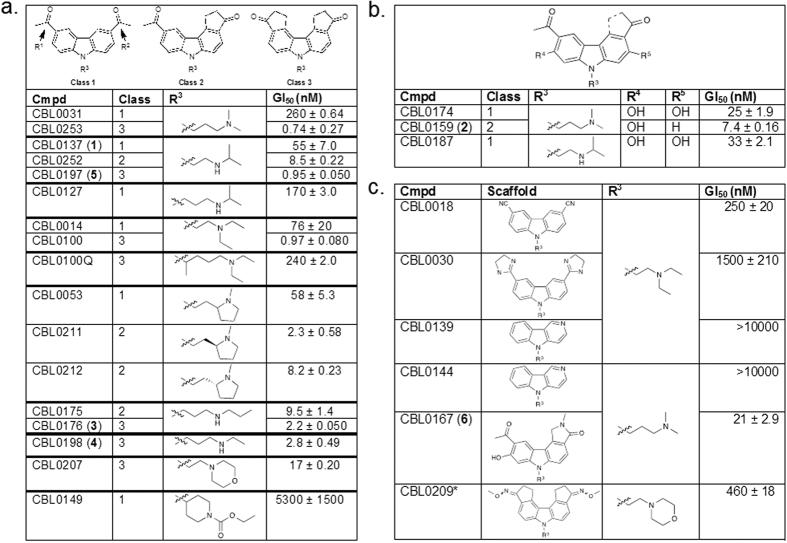
Inhibition of *T*. *brucei* proliferation: Exploratory Structure-Activity Relationship (SAR). *T*. *brucei* (4 × 10^3 ^cells/mL) in 24-well or 96-well plates were incubated with DMSO or compound (various concentrations) for 48 h. The amount of drug that inhibits trypanosome proliferation 50% (GI_50_) was determined for each compound. Mean GI_50_ were determined from two independent experiments (totaling four separate biological replicates) within +/− standard deviation. (**a**) SAR of Class 1 (R^1^ and R^2^ = acetyl groups), Class 2 (R^1^ = acetyl group, R^2^ = cyclopentanone), and Class 3 (R^1^ and R^2^ = cyclopentanone). (**b**) Class 1, 2 or 3 structures with hydroxyls at R^4^ and/or R^5^. (**c**) Other compounds not qualified as Class 1, 2 or 3. *For CBL0209 we do not know which E/Z isomers were tested.

Twenty-six compounds were screened for their effects on trypanosome proliferation *in vitro*, by culturing bloodstream *T*. *brucei* in different concentrations of each compound. Test compound concentrations causing 50% inhibition of trypanosome proliferation (GI_50_) were calculated (Fig. 1, Supplementary Fig. S1). Compounds with GI_50_ less than 100 nM were classified as “hits”^36^.

Most compounds were categorized into one of three classes according to the R^1^ and R^2^ substituents on the carbazole scaffold (Fig. 1a,b). The most potent inhibitors belonged to Class 3, containing two cyclopentanone rings fused with the carbazole. Most of these inhibitors displayed a GI_50_ between 0.7–3 nM. Class 2 compounds (with one fused cyclopentanone ring and one acetyl group) had GI_50_ between 2–10 nM. Class 1 compounds (diacetyl-substituted on the carbazole) had GI_50_ between 25–300 nM. We could hypothesize a few reasons why this conformational restraint may be relevant. First, this ring restraint could reinforce presentation of the hydrogen bond acceptor carbonyl(s) in a vector that made more favorable interactions with its target(s) of action. Second, the enhanced planarity compared to the acyclic acetyl groups could enhance DNA intercalation. Lastly, we do not rule out a possibility that this structural feature affects other properties that contributed to the potency of Class 3 inhibitors against the parasite. Work is ongoing to test these hypotheses.

Other compounds that did not cleanly fit into the classification system above were tested (Fig. 1c). These compounds all presented H-bond acceptor moieties that could potentially mimic the cyclopentanone carbonyl oxygen, however only one, CBL0167 (**6**), had appreciable activity with GI_50_ less than 25 nM (Fig. 1c).

With the exception of CBL0149 (Fig. 1a), the *N*-linked side chain of each compound tested contained either a basic secondary or tertiary amine. Within the Class 1 compounds, increasing chain length and steric bulk around the amine generally reduced potency. For example, extension of the chain from two to three carbons (**1** vs. CBL0127, Fig. 1a) resulted in a 3-fold reduction in potency. In another example, increasing the steric bulk on the linker (CBL0100Q versus CBL0100, Fig. 1a) led to a nearly 250-fold potency loss. Lastly, we noted that hydroxylation of the carbazole core was tolerated in three examples (such as compound **2**, Fig. 1b). This initial SAR was instructive with regards to the first round of medicinal chemistry optimization, which will be reported in due course.

### Selection of compounds for testing in a mouse model of HAT

To explore anti-trypanosomal effects of the compounds in mice, we selected representatives of each class as defined in Fig. 1. Compound **1** was the best Class 1 candidate to test *in vivo* because it is well-tolerated in mice following oral administration^17^, and it had a low GI_50_ of 55 nM against *T*. *brucei* (Fig. 1a). Compounds **2** (Class 2) and **3** (Class 3) were chosen because they were also orally bioavailable (Cleveland BioLabs, unpublished data) and they had low GI_50_’s of 7.4 nM and 2.2 nM respectively. Furthermore, compounds **1**–**3** were potent against the human-infective subspecies *T*. *brucei rhodesiense* (Table 1).

Prior to performing drug efficacy studies in a mouse model of HAT, we sought to identify overtly toxic compounds, by determining the GI_50_ of compounds **1**–**3** against HeLa cells *in vitro* (Table 1). The selectivity index (SI) for each compound was calculated as a ratio between HeLa and *T*. *brucei* GI_50_ values. Because **1** is well-tolerated in mice^17^, it’s SI (38-fold) was used as a threshold for compound selection. Both **2** and **3** had SI values greater than that of **1** (SI = 78 and 236-fold, respectively), indicating less general toxicity by these criteria. Therefore, **1** (Class 1), **2** (Class 2) and **3** (Class 3) were selected for testing in a mouse model of HAT.

### Compound 2 and Compound 3 extend life of mice infected with *T*. *brucei*

**Figure 2:**
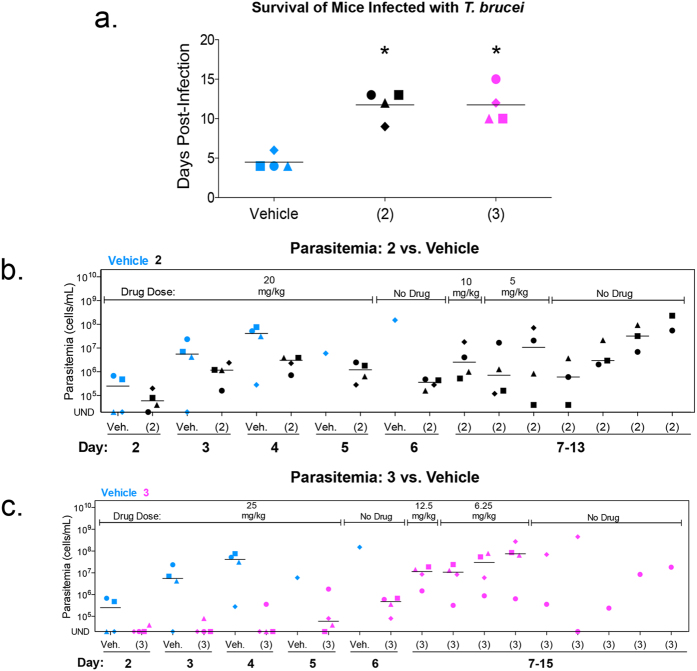
Compounds 2 and 3 reduce trypanosome proliferation in mice. Mice (n = 4 per group) were infected intraperitoneally with 1 × 10^4^ bloodstream *T*. *brucei*. Compound **2**, **3** and vehicle were administered orally as indicated in graphs. Doses administered of compound **2**: 5 mg/kg, 10 mg/kg and 20 mg/kg. Doses administered of compound **3**: 6.25 mg/kg, 12.5 mg/kg and 25 mg/kg (**a**) Mean survival of vehicle and compound treated mice. Horizontal lines indicate mean survival (days). Student’s *t*-test was used to compare survival of vehicle to drug-treated mice. *P < 0.0007. (**b**,**c**) Parasitemia of **2** (**b**) or **3** (**c**) treated mice compared to vehicle. Individual mice are represented by different symbol shapes. Horizontal lines indicate median parasitemia.

Following infection with trypanosomes, mice from the vehicle (*i*.*e*., untreated) control group survived 5 days on average. Mice treated orally with **2** or **3** had a 2-fold increase in average survival (Fig. 2a). From days two to five a full dose (at the repeated maximum tolerated dose) of either **2** or **3** was orally administered to mice (20 mg/kg and 25 mg/kg respectively). At day six, both compounds reduced parasitemia 100-fold (Fig. 2b,c). However, weight loss exceeding 10% of normal body weight (NBW) was observed in both treatment groups (Supplementary Fig. S2b,c). As a result, over the next several days, doses of both compounds were reduced to limit weight loss. After treatment with **2** and **3** ended (day 10), parasitemia rose and all mice were euthanized for humane reasons because of high parasitemia (>2 × 10^8 ^cells/mL) (Fig. 2b,c).

### Compound 1 cures *T*. *brucei* infection in a mouse model of HAT

**Figure 3:**
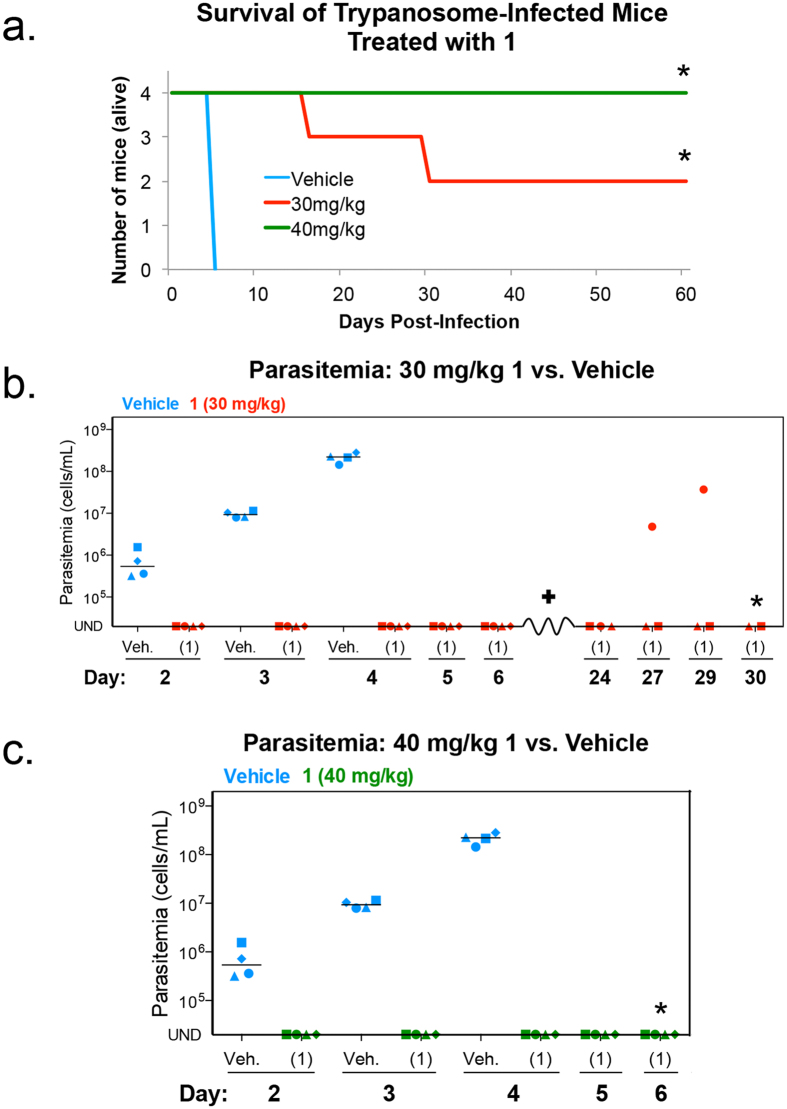
Compound 1 cures *T*. *b*. *brucei* infection in a mouse model of HAT. Mice (n = 4 per group) were infected intraperitoneally with 1 × 10^4^ bloodstream *T*. *brucei*. Compound **1** (30 mg/kg or 40 mg/kg) and vehicle were administered orally once per day of treatment for a total of 14 doses. (**a**) Number of mice alive post-infection. *All remaining mice were cured of trypanosome infection. (**b**,**c**) Parasitemia in mice dosed with vehicle or 30 mg/kg of compound **1** (**b**) or 40 mg/kg of compound **1** (**c**). Individual mice are represented by different symbol shapes. Horizontal lines indicate median parasitemia. UND = parasitemia undetectable. Mouse (♦) died on day 16 post-infection, no parasitemia observed prior to death (data not presented). *All remaining mice were cured of trypanosome infection.

Compound **1** was administered to *T*. *brucei*-infected mice once per day of treatment (4-on, 2-off) at 30 mg/kg or 40 mg/kg for a total of 14 doses. Little or no weight loss was observed during treatment with **1** (Supplementary Fig. S2a). Vehicle control mice were euthanized for humane reasons by day five because their parasitemia was greater than the 2 × 10^8 ^parasites/mL threshold. All mice treated with **1** were alive on day five. A 30 mg/kg dose of **1** cured 50% of trypanosome-infected mice. Following administration of 40 mg/kg, 100% of trypanosome-infected mice were cured of infection (Fig. 3a). Trypanosomes were not observed in peripheral blood of infected mice at either dose level (Fig. 3b,c). Of the two mice that died during the 30 mg/kg treatment, one had no parasitemia and may have died of other causes, whereas the second mouse died following recrudescence observed on day 27 post-infection (Fig. 3b). Overall, this data demonstrated efficacy of **1** in a mouse model of HAT, and established **1** as a lead for *T*. *brucei* drug development.

### Compound 1 affects DNA replication and mitosis

Following discovery of **1** as a lead, we explored that drug’s mode of action by examining its effect on trypanosome cell division. Trypanosomes have chromosomal DNA in the nucleus, and their mitochondrial DNA (kinetoplast DNA (kDNA)) is found in a nucleoid termed the kinetoplast within the mitochondrion. Because compound **1** binds DNA in HeLa cells^17^ we compared its effects on trypanosome biology to that of ethidium bromide (EtBr), a well-known DNA binding agent that is also anti-trypanosomal^37^.

**Figure 4:**
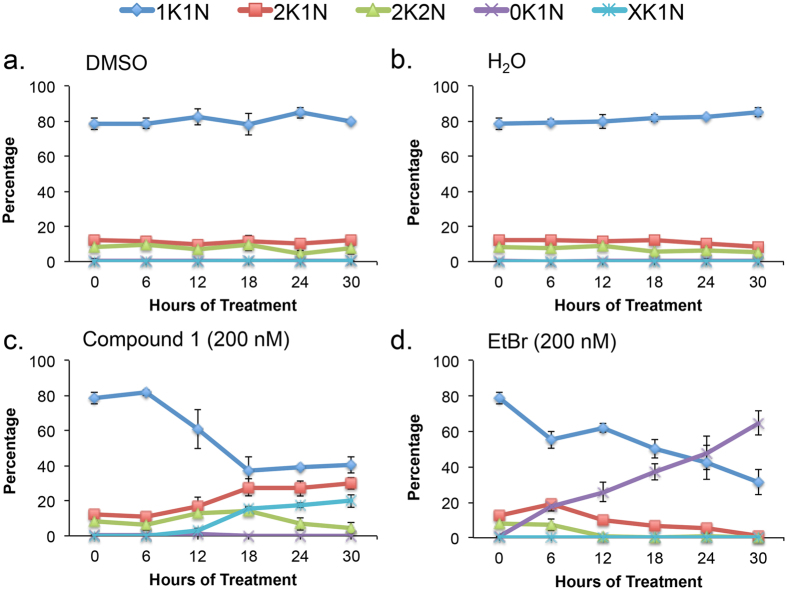
Summary of effects of compound 1 and ethidium bromide on trypanosome nucleus and kinetoplast copy number. Results summarized from Supplementary Fig. S5. (**a**) DMSO-treated, (**b**) H_2_O-treated, (**c**) **1**-treated, (**d**) ethidium bromide-treated. Mean percentage of cells +/− standard deviation were determined from three independent experiments.

**Figure 5:**
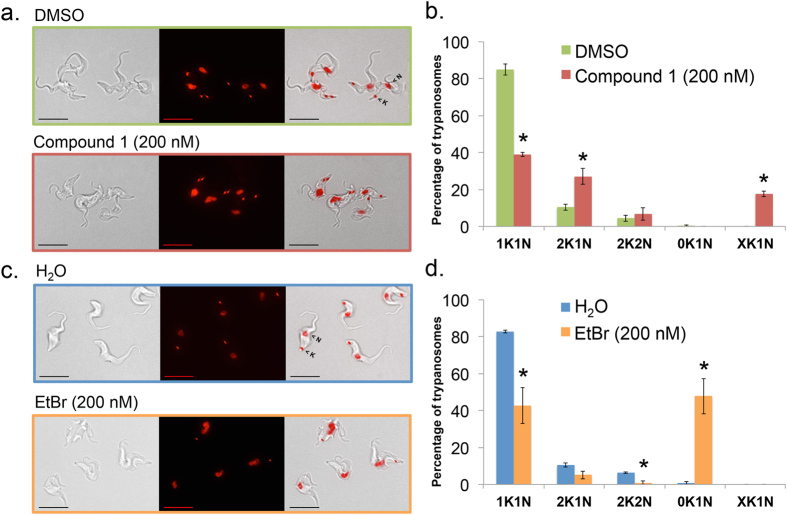
Effects of 24 h compound 1 treatment on trypanosome nucleus and kinetoplast copy number. *T*. *brucei* (1 × 10^5 ^cells/mL) were treated with **1** (200 nM), ethidium bromide (200 nM), H_2_O, or DMSO (0.1% vol/vol) for 24 h as part of a 30 h time-course in HMI-9 medium (Supplementary Fig. S5). Cells were fixed with paraformaldehyde (4% in PBS) and DNA was stained with DAPI (1.5 μM). (**a**,**c**) Representative images of 24 h treated cells. Panels = Left: DIC (differential interference contrast), middle: DAPI (red), right: Merge. Bar = 10 μm. (**b**,**d**) Nuclei (N) and kinetoplasts (K) were counted from 150 cells for each sample. 1K1N, trypanosomes with one kinetoplast/one nucleus; 2K1N, trypanosomes with two kinetoplasts/one nucleus; 2K2N, trypanosomes with two kinetoplasts/two nuclei; 0K1N, trypanosomes without visible kinetoplasts/one nucleus; XK1N, trypanosomes with more than two kinetoplasts/one nucleus. Mean percentage of cells +/− standard deviation were determined from three independent experiments. Student’s *t*-test was used to compare organelle copy number distribution of vehicle (DMSO or H_2_O) to drug-treated trypanosomes. *P < 0.05, determined by Student’s *t*-test.

**Figure 6:**
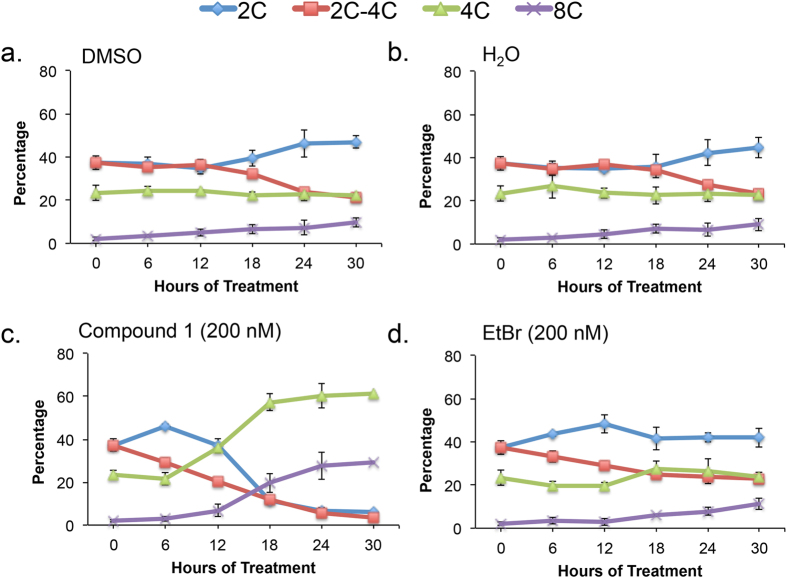
Summary of effects of compound 1 and ethidium bromide on nuclear DNA content. Results summarized from Supplementary Figs S7 and S8. (**a**) DMSO-treated, (**b**) H_2_O-treated, (**c**) **1**-treated, (**d**) ethidium bromide-treated. Mean percentage of cells +/− standard deviation were determined from three independent experiments.

**Figure 7:**
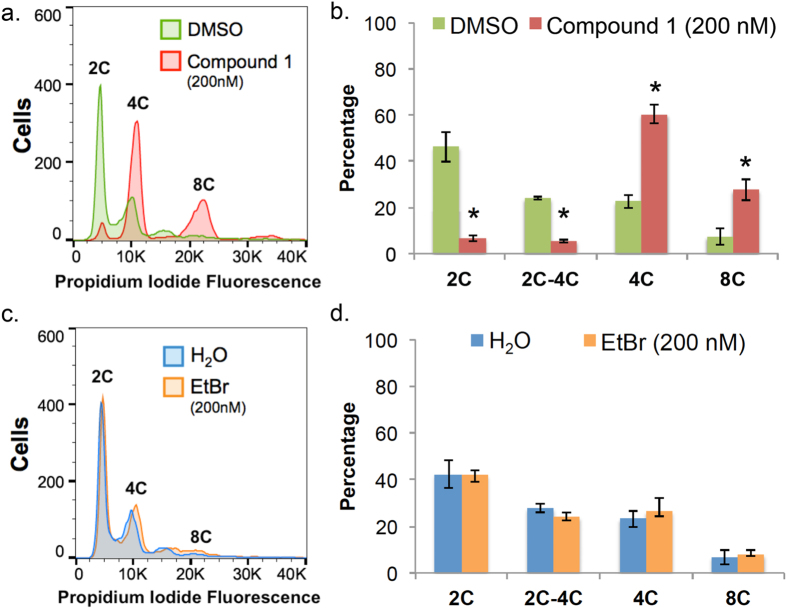
Effects of 24 h compound 1 treatment on nuclear DNA content. *T*. *brucei* (10^5 ^cells/mL) were treated with **1** (200 nM), ethidium bromide (200 nM), H_2_O, or DMSO (0.1% vol/vol) for 24 h as part of a 30 h time-course in HMI-9 medium (Supplementary Figs S7 and S8). Cells were fixed with PBS containing 70% methanol, treated with RNase A (500 μg/mL) and DNA was stained with propidium iodide (7.5 μM). (**a**,**c**) Histograms of DNA content per cell. 10,000 trypanosomes were analyzed per sample. Chromosomal content (e.g. “2C”) is indicated for each peak. (**b**,**d**) Proportion of cells with DNA content from 2C–8C. Mean percentage of cells +/− standard deviation were determined from three independent experiments. *P < 0.05, determined by Student’s *t*-test comparing DNA content of vehicle (DMSO or H_2_O) to drug-treated trypanosomes.

A 30 h time-course of ethidium bromide (EtBr, 200 nM) and **1** (200 nM) treatment was performed with their appropriate vehicle controls under conditions reported to cause dyskinetoplastic formation during EtBr treatment^37^. Cell density and effects on DNA “karyotype” (*i*.*e*., number of nuclei and kinetoplasts per cells) and DNA content were determined from samples collected every 6 h (Figs 4, 5, 6 and 7, Supplementary Figs S4–S8). Both compounds blocked trypanosome proliferation, with only 1.5-fold and 2.5-fold increases in cell density during 30 h treatment with **1** or ethidium bromide (Supplementary Fig. S4). Generally, effects on DNA karyotype/content by either drug reached maximum effect by 24 h (Figs 4 and 6). Therefore, representative microscopy images presented were taken only from trypanosomes obtained at this time-point (Fig. 5 and Supplementary Fig. S6).

Ethidium bromide (200 nM) produced dyskinetoplastic trypanosomes (*i*.*e*., cells that lack a kinetoplast but contain a nucleus, 0K1N) within 6 h (Fig. 4d). Proportions of dyskinetoplastic trypanosomes increased whereas proportions of cells with one kinetoplast one nucleus (1K1N) and cells with two kinetoplasts and one nucleus (2K1N) decreased during the 30 h treatment (Figs 4d and 5c,d). Compound **1** (200 nM) failed to generate dyskinetoplastic trypanosomes. Instead, **1** increased the fraction of 2K1N cells while decreasing the proportion of 1K1N cells. Furthermore, **1** caused the formation of XK1N trypanosomes containing three or four kinetoplasts and one nucleus (Figs 4c and 5a,b and Supplementary Fig. S6). These observations indicated that **1** inhibits mitosis, but did not affect kinetoplast biogenesis. These differences in the biological effects of EtBr and **1** suggested that the targets of the two compounds are different.

Effects of 30 h treatment with **1** (200 nM) or EtBr (200 nM) on trypanosome DNA content were analyzed (Figs 6 and 7 and Supplementary Figs S7 and S8). For **1**, most treated cells contained 4C DNA and a significant fraction (~30%) were polyploid with 8C DNA (Figs 6c and 7a,b and Supplementary Fig. S7). These data indicated that although **1**-treated trypanosomes failed mitosis they could, surprisingly, re-enter S-phase of the cell division cycle. Ethidium bromide caused little or no effect on nuclear DNA content compared to the H_2_O (vehicle) control (Figs 6d and 7c,d and Supplementary Fig. S8).

### Compound 1 has a “delayed killing” effect on *T*. *brucei*

**Figure 8:**
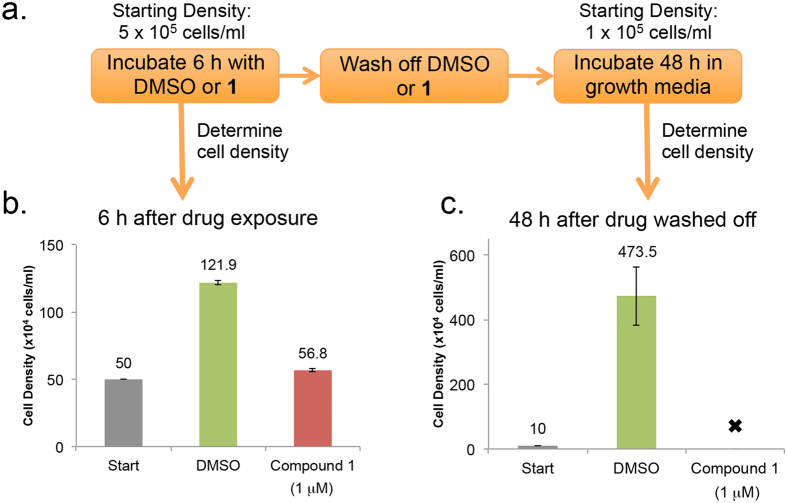
Delayed killing effects of Compound 1 on *T*. *brucei*. *T*. *brucei* (5 × 10^5 ^cells/mL) were treated with 1 (1 μM) or DMSO (0.1% vol/vol) for 6 h. Cells were washed twice with HMI-9 medium, resuspended at 1 × 10^5 ^cells/mL, and cultured for 48 h. Trypanosome densities were determined with a hemocytometer. (**a**) Flow chart summarizing trypanosome treatment and recovery protocol prior to analysis. (**b**) Cell density before (“Start”) and after 6 h treatment with DMSO or **1** (1 μM). (**c**) Cell density after wash and resuspension at 1 × 10^5 ^cell/mL (“Start”) and after 48 h incubation in drug-free medium. Mean cell density (values above each bar) ± standard deviation is presented from two independent experiments, four separate biological replicates. “” indicates no cells observed.

Compound **1** cured trypanosome infection in the mouse model of acute HAT (Fig. 3) whereas 30 h incubation with 200 nM of the drug arrested proliferation of *T*. *brucei in vitro* (Supplementary Fig. S4). To explain these data, we hypothesized that exposure of trypanosomes to **1** could lead to “delayed killing” of the cells after removal of drug from culture medium. To test this theory, an experiment was designed to best mimic conditions of detectable parasitemia and reasonable drug exposure in a mouse infection. We used 5 × 10^5 ^trypanosomes/mL and 1 μM of compound **1**, because the maximum concentration of **1** observed in mouse blood plasma (C_max_) was 2.25 μM after a single oral dose of 30 mg/kg (Supplementary Fig. S3 and Supplementary Table S1). Trypanosomes were treated with **1** (1 μM) for 6 h after which the drug was washed off, trypanosomes were seeded in fresh medium and incubated for 48 h, eight division cycles (Fig. 8).

Following 6 h compound **1** treatment, wash off, and 48 h incubation in drug-free medium, control cells treated with DMSO proliferated, whereas all **1**-treated trypanosomes died (Fig. 8b,c). We concluded that trypanosomes are damaged irreversibly within hours of exposure to **1**. Delayed killing effects were restricted to trypanosomes because no such effects on HeLa proliferation were observed under similar experimental conditions (Supplementary Fig. S9).

## Discussion

Drugs currently used to treat HAT are in great need of improvement^1^. Unfortunately, because HAT is a disease of poverty, discovering better drugs against the disease is not suited for expensive research by major pharmaceutical companies^38^. In this study, we employed a “drug repurposing” strategy^7^,^8^ to establish **1** as a lead for anti-trypanosome drug discovery.

By comparing compounds with matching substituents at R^3^, R^4^, and/or R^5^ we observed that replacing cyclopentanones with acetyl groups or other chemical structures at R^1^ or R^2^ always reduced the anti-trypanosomal activity of the compound (*e*.*g*. compare **5** to CBL0252 to **1**, Fig. 1a). Overall, these observations supported the significance of a rotationally-restricted cyclopentanone ring in enhancing anti-trypanosomal features. Cyclopentanone rings provided an electron-withdrawing effect, a hydrogen-bond acceptor, hydrophobicity, and steric restriction. Restraint of the rotation posed by the cyclopentanone ring may have placed the carbonyl group oxygen atom(s) at an ideal orientation for better interaction with the compound’s target(s) of action, when compared to the Class 1 and 2 analogs. Finally, the importance of R^1^ and R^2^ on drug potency was illustrated best by CBL0139 and CBL0144 (Fig. 1c). Both compounds contained a nitrogen within the carbazole ring system with no attached substituent, and they were both inactive (GI_50_ > 10 μM) against *T*. *brucei*.

The use of proliferation inhibition assays for hit discovery (Supplementary Fig. S1) is standard for identifying anti-trypanosomal compounds^26^,^30^,^39^. Moreover, there has been a recent emphasis on identifying compounds that kill *T*. *brucei* rather than inhibit proliferation^40^. However, as our work suggests, proliferation inhibition is not always the best indicator of drug efficacy in the mouse model of HAT. For example, **2** and **3** had excellent GI_50_ values that were less than 10 nM (Table 1). However, **2** and **3** failed to cure *T*. *brucei* infection in mice (Fig. 2). Conversely, **1** had relatively less impressive anti-proliferative activity (Table 1). Yet it was **1** that cured a trypanosome infection in mice (Fig. 3). One possible explanation for these results is that varying toxicity of different analogs in mice limits how much drug can be administered to clear the infection without killing the mouse (Supplementary Fig. S2).

Studies investigating the mode of action of our lead compound, **1**, revealed that the principal biological effect was inhibition of mitosis (Figs 4 and 5, Supplementary Figs S5 and S6). However, it is not possible to completely exclude other effects contributing to trypanosome death.

Compound **1** treatment led to a build-up of 2K1N and XK1N cells (Fig. 4c) and blocked trypanosome proliferation (Supplementary Fig. S4). The population of cells with 2C DNA was depleted, and instead most trypanosomes had 4C and 8C DNA (Fig. 6c). Together, these data indicated that trypanosomes were unable to execute mitosis, failed to undergo cytokinesis and they re-entered S-phase marked by (i) synthesis of chromosomal DNA, and (ii) production of new kinetoplasts. Consistent with these concepts, detection of trypanosomes with 8C DNA content was concurrent with appearance of XK1N at 18 h (Figs 4c and 6c).

Mitosis inhibition accompanied by ongoing DNA synthesis (both nuclear and mitochondrial) has been reported in bloodstream form (BSF) *T*. *brucei* following knockdown of select protein kinases or cyclins^41^,^42^. Although compound **1** is a non-genotoxic DNA intercalator in a human cell^17^, we failed to detect it in trypanosome nuclei using the published conditions. However, the ability to inhibit mitosis and not DNA replication argues against compound **1** principally acting as a DNA intercalator in *T*. *brucei*. Instead, compound **1** could target proteins. Compound **1** is a carbazole, some of which bind proteins^12^,^16^,^17^,^18^,^43^,^44^,^45^,^46^,^47^,^48^,^49^,^50^. So it is possible that compound **1** has a protein target in trypanosomes.

Three protein kinases (Cdc-2 related kinase 3 (CRK3), Aurora kinase 1 (AUK1) and tousled-like kinase 1 (TLK1)) are of interest as possible targets of **1** because they regulate both nuclear DNA replication and mitosis. Knockdown of CRK3^51^, AUK1^52^ or TLK1^53^ in bloodstream *T*. *brucei* results in build-up of 2K1N cells with 4C DNA content. Most strikingly, sustained knockdown of AUK1 led to an accumulation of trypanosomes with 8C DNA content, a single enlarged nucleus, and multiple kinetoplasts similar to the XK1N trypanosomes observed after **1** treatment (Supplementary Fig. S6). CRK3 and TLK1 knockdown also produced an XK1N population, however, effects on DNA content above 4C were not reported.

Mitotic arrest preceding overreplication of DNA has been observed in select vertebrate cells either as a natural process (*i*.*e*., differentiation) or induced by drug treatment. In (i) “mitotic slippage” or (ii) endoreplication, human glioma cells treated with nocodazole^54^, or megakaryocytes undergoing endoreplication^55^,^56^,^57^,^58^, arrest at mitosis but continue to synthesize DNA.

In summary, we have found that the carbazole compound **1** is an orally bioavailable lead for anti-trypanosome drug discovery, and established its mode of action: it blocks mitosis but allows licensing of chromosomal and kinetoplast DNA replication. Carbazoles such as carprofen and other carbazole derivatives are being evaluated as leads for drugs to treat human neurodegenerative disorders^12^,^13^,^14^,^15^,^16^. Additionally, some carbazole derivatives are active against other protozoan parasites, such as *Leishmania donovani*^59^, and *Plasmodium falciparum*^60^,^61^. Thus, carbazoles are a promising chemical scaffold for “repurposing” in an effort to discover new and better anti-trypanosomal drugs.

## Additional Information

**How to cite this article**: Thomas, S. M. *et al*. Discovery of a Carbazole-Derived Lead Drug for Human African Trypanosomiasis. *Sci. Rep.* **6**, 32083; doi: 10.1038/srep32083 (2016).

## Supplementary Material

Supplementary Information

## Figures and Tables

**Table 1:** Selectivity Index of hits.

Cmpd	Growth inhibition (GI_50_) ± SD (nM)			Selectivity Index
	*T*. *b*. *rhodesiense*	*T*. *b*. *brucei*	*HeLa*	*HL GI*_*50*_/*Tbb GI*_*50*_
1	58 ± 3	55 ± 7	2100 ± 50	38
2	13 ± 3	7.4 ± 0.2	580 ± 100	78
3	3.6 ± 0.3	2.2 ± 0.1	520 ± 40	236

Proliferation inhibition of compounds against *T*. *b*. *brucei*, *T*. *b*. *rhodesiense* and human HeLa (HL) cells. *T*. *brucei* (4 × 10^3 ^cells/mL) in 24-well plates were incubated with DMSO (0.1% vol/vol) or compound (various concentrations) for 48 h. The amount of drug that inhibits trypanosome proliferation 50% (GI_50_) was determined for each compound. Mean GI_50_ ± standard deviation was determined from two independent experiments, four separate biological replicates.
