# Erratum: Discovery of a Carbazole-Derived Lead Drug for Human African Trypanosomiasis

**DOI:** 10.1038/srep40565

**Published:** 2017-01-11

**Authors:** Sarah M. Thomas, Andrei Purmal, Michael Pollastri, Kojo Mensa-Wilmot

Scientific Reports 6: Article number: 32083; 10.1038/srep32083 published online: 08 26 2016; updated: 01 11 2017.

In this Article, Andrei Purmal is incorrectly listed as being affiliated with ‘Cleveland BioLabs, Inc., Buffalo, New York 14203, USA’. The correct affiliation is listed below:

Incuron LLC, Buffalo, NY, 14203.

