# Supplementary material for: Discovery of a Carbazole-Derived Lead Drug for Human African Trypanosomiasis
Source: Sci Rep. 2016 Aug 26;6:32083. doi: 10.1038/srep32083 (PMC5000474; doi:10.1038/srep32083)
Supplement: Supplementary Information [file srep32083-s1.docx]

**Supplementary Figures**

**DISCOVERY OF A CARBAZOLE-DERIVED LEAD DRUG FOR HUMAN AFRICAN TRYPANOSOMIASIS**

Sarah M. Thomas, Andrei Purmal, Michael P. Pollastri, and Kojo Mensa-Wilmot.

**Figure S1. Inhibition of *T. brucei* proliferation *in vitro*.** *T. brucei* (4 x 10^3^ cells/mL) in 24-well or 96-well plates were incubated with DMSO or compound (various concentrations) for 48 h. The amount of drug that inhibits trypanosome proliferation 50% (GI_50_) was determined for each compound. Mean GI_50_ +/- standard deviation were determined from two independent experiments, four separate biological replicates.

**Figure S2. Drug effects on normalized body weight (NBW) in *T. brucei* infected mice.** Mice (n = 4 per group) were infected intraperitoneally with 10^4^ bloodstream *T. brucei*. Compound **1**, **2**, **3** and vehicle were administered orally starting 1 day post-infection. **a**. Average NBW during (**a)** compound **1**, (**b)** compound **2** or **(c)** compound **3** *in vivo* efficacy studies. Compound **1** (30 mg/kg or 40 mg/kg) and vehicle were administered orally once daily for a total of 14 doses, 4-on/2-off regimen. Doses (mg/kg) and days of administration are indicated in graphs for compound **2** **(b)** and compound **3** **(c)**. Average NBW per group ± standard deviation is presented.

**Table S1. Pharmacokinetic parameters of Compound 1 in mice following single 30 mg/kg dose.** CD-1 female mice were administered a single oral (gavage) or intravenous (slow bolus) dose of 30 mg/kg of **1**. Blood samples (approximately 0.5 mL) were collected from the orbital sinus of mice. Each mouse was used for blood collection at one time point only, with a total of four mice at each collection time point. Mouse blood collected at each time point was processed to prepare plasma samples. Plasma samples were analyzed using an LC/MS/MS method to determine CBL0137 concentration. The concentration of compound **1** was determined in samples collected at 0 (pre-dose), 2.5, 5, 15, 30 minutes and 1, 2, 4, 6, 8, 16, 24, and 32 hours following oral or IV dosing. Pharmacokinetic parameters (maximal plasma concentration, **C_max_**; area under the concentration-time curve, **AUC_0→Cp_**; and terminal elimination half-life (**t_1/2λz_**) were calculated using a model-independent approach (non-compartmental analysis) using a uniform weighting scheme with WinNonlin version 6.2 (Pharsight, Cary, NC). Area under the concentration-time curve (AUC_0-Cp_) from time zero (0) to the last measured time point (Cp) was determined using the linear up/log down trapezoidal rule. The area was extrapolated to infinity (**AUC_0-∞_**) using the rate constant of the terminal elimination phase (**λ_z_**). λ_z_ was determined from the slope of the terminal log-linear portion of the concentration-time curve using the last three measureable time points after the C_max_. The elimination half-life (**t_1/2λz_**) was calculated by dividing λ_z_ into the natural logarithm of 2. Peak plasma concentration (**C_max_**) and the time of C_max_ (**t_max_**) were determined directly from the individual observed concentration-time data. The concentration at time zero (**C_0_**) following IV administration was determined by back-extrapolation of the initial concentration-time points. Clearance (**CL**) and Volume of Distribution (**V_z_**) were calculated by the following equation:

The absolute bioavailability (**F**) of oral administration of CBL0137 was calculated using the following equation:


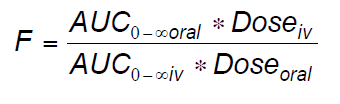

**Figure S3. Mean plasma concentration-time profile following single 30 mg/kg dose of CBL0137**. Mean plasma concentration of CBL0137 was determined during the initial absorption and elimination within 4 hours of administration (**a**) or over the 32-hour blood collection period (**b**) for both oral and IV routes.

**Figure S4. Effects of compound 1 on trypanosome proliferation.** *T. brucei* (10^5^ cells/mL) were treated with **1** (200 nM), ethidium bromide (200 nM), H_2_O, or DMSO (0.1% vol/vol) for 30 h in HMI-9 medium. Cell density was determined every 6 h using a Z2 Coulter Counter. **(a)** Cell density of DMSO and **1**-treated samples. **(b)** Cell density of H_2_O and ethidium bromide treated samples. Mean cell density ± standard deviation is presented from three independent experiments. Mean percentage of cells +/- standard deviation were determined from three independent experiments. Student’s *t*-test was used to compare cell density of vehicle (DMSO or H_2_O) to drug-treated trypanosomes at each time-point. * *P* < 0.05, determined by Student’s *t*-test.

**Figure S5. Effects of compound 1 and ethidium bromide on trypanosome nucleus and kinetoplast copy number.** *T. brucei* (10^5^ cells/mL) were treated with **1** (200 nM), ethidium bromide (200 nM), H_2_O, or DMSO (0.1% vol/vol) for 30 h in HMI-9 medium. Aliquots of treated cells were collected every 6 h, fixed with paraformaldehyde (4% in PBS) and stained with DAPI (1.5 μM). Quantitation of nuclei (N) and kinetoplasts (K) counted from 150 cells for each sample. **(a)** DMSO vs. **1**. **(b)** H_2_O vs. ethidium bromide. Mean percentage of cells +/- standard deviation were determined from three independent experiments. Student’s *t*-test was used to compare organelle copy number distribution of vehicle (DMSO or H_2_O) to drug-treated trypanosomes. * *P* < 0.05, determined by Student’s *t*-test.

**Figure S6. “XK1N” trypanosomes observed after 24 h compound 1 treatment.** *T. brucei* (10^5^ cells/mL) were treated with **1** (200 nM), or DMSO (0.1% vol/vol) for 24 h in HMI-9 medium. Cells were fixed with paraformaldehyde (4% in PBS) and DNA was stained with DAPI (1.5 μM). Representative images of 24 h treated trypanosomes **(a)** DMSO or **(b)** compound **1**. Panels = Left: DIC (differential interference contrast), middle: DAPI (red), right: Merge. Bar = 10 μm. N= nucleus, K= kinetoplast.

**Figure S7. Effects of compound 1 on nuclear DNA content.** *T. brucei* (10^5^ cells/mL) were treated with **1** (200 nM) or DMSO (0.1% vol/vol) for 30 h in HMI-9 medium. Aliquots of treated cells were collected every 6 h, fixed with PBS containing 70% methanol, treated with RNase A (500 μg/mL) and DNA was stained with propidium iodide (7.5 μM). **(a)** Histograms of DNA content per cell (DMSO vs. **1**). 10,000 trypanosomes were analyzed per sample. Chromosomal content (e.g. “2C”) is indicated for each peak. **(b)** Proportion of cells with DNA content from 2C – 8C. All values were determined with FlowJo software. Mean percentage of cells +/- standard deviation were determined from three independent experiments. * *P* < 0.05, determined by Student’s *t*-test.

**Figure S8. Effects of ethidium bromide on nuclear DNA content.** *T. brucei* (10^5^ cells/mL) were treated with ethidium bromide (200 nM) or H_2_O for 30 h in HMI-9 medium. Aliquots of treated cells were collected every 6 h, fixed with PBS containing 70% methanol, treated with RNase A (500 μg/mL) and DNA was stained with propidium iodide (7.5 μM). **(a)** Histograms of DNA content per cell (H_2_O vs. EtBr). 10,000 trypanosomes were analyzed per sample. Chromosomal content (e.g. “2C”) is indicated for each peak. **(b)** Proportion of cells with DNA content from 2C – 8C. All values were determined with FlowJo software. Mean percentage of cells +/- standard deviation were determined from three independent experiments. * *P* < 0.05, determined by Student’s *t*-test.

**Figure S9. Delayed effects of Compound 1 on HeLa cell proliferation.** HeLa cells (1 x 10^5^ cells/mL) were incubated in wells of a 24-well plate for 24 h then treated with **1** (1 μM) or DMSO (0.1% vol/vol) for 6 h. Cells were washed and resuspended in equivalent volumes of HMI-9 medium. Cell density was determined using a Neubauer Bright-line hemacytometer as described in Methods. Samples were resuspended in HMI-9 medium to 5 x 10^4^ cells/mL and incubated for 48 h. Cells were counted and resuspended every 48 h until 8 days (192 h) post-treatment. Mean cell density ± standard deviation is presented from two independent experiments, four separate biological replicates. Vertical connections in graph represent a resuspension down to 5 x 10^4^ cells/mL. Differences in cell density between control and experimental samples were analyzed by Student’s *t*-test. No significant statistical differences were detected, all *p*-values > 0.1.
